# Exploiting paracrine mechanisms of tissue regeneration to repair damaged organs

**DOI:** 10.1186/2047-1440-2-10

**Published:** 2013-06-20

**Authors:** Diana F Anthony, Paul G Shiels

**Affiliations:** 1 Department of Surgery, Institute of Cancer Sciences, College of Medical, Veterinary and Life Sciences, University of Glasgow, Western Infirmary, Glasgow G11 6NT, UK

**Keywords:** Stem cells, Paracrine action, Tissue and cell regeneration, Microvesicles

## Abstract

Stem cells have been studied for many years for their potential to repair damaged organs in the human body. Although many different mechanisms have been suggested as to how stem cells may initiate and facilitate repair processes, much remains unknown. Recently, there has been considerable interest in the idea that stem cells may exert their effects *in vivo* via paracrine actions. This could involve the release of cytokines, growth factors or secreted extracellular vesicles. This article reviews the role that paracrine actions may play in tissue regeneration. In particular, it considers how microvesicles, as a mediator or modulator of paracrine action, can be exploited as a tool for non-cell-based therapies in regenerative medicine.

## Introduction

The potentially advantageous effects of using stem cells in tissue regeneration were first demonstrated by Till and McCulloch in the 1960s [1,2]. This pivotal discovery paved the way for regenerative medicine, which in the last decade has advanced significantly. Regenerative medicine is broadly defined as the study of the repair, replacement, regeneration and restoration of diseased, damaged or aged cells, tissues or organs [3,4]. It has emerged as a serious solution to the repair of end-stage organ damage and to address the growing shortfall in donor organs for transplantation. Its clinical use is still very much in its infancy and much remains to be determined about the mechanisms of action for cellular therapeutics. In this review, we will attempt to summarise some of the current state-of-the-art information available in this field, with an emphasis on mesenchymal stem cells and other non-embryo-derived cellular therapies, which are advancing rapidly towards the clinic, with special reference to paracrine-mediated tissue repair.

**Figure 1:**
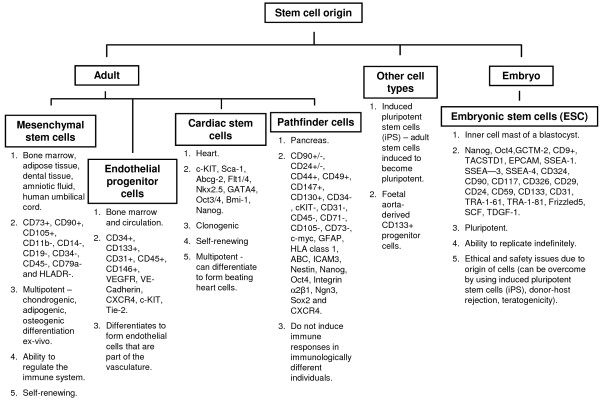
**Overview of the general properties of stem cell types.** The chart shows the different types of available stem cells and the surface markers used to identify cells of both adult and embryonic origin as well as other stem cell types.

The first successful demonstration of regenerative medicine, using a cell-based therapy to repair damaged tissue, was described by Ferrari *et al*. [5]. This group showed that regeneration of damaged muscle fibres was possible by transplanting bone marrow stem cells into injured muscle tissue [5]. Subsequently, a variety of different stem cell types has been isolated and investigated for use in tissue regeneration, with varying degrees of success. These include mesenchymal stem cells (MSCs) [6-9], adipose-derived stem cells (ASCs), also known as adipose-derived MSCs [10], embryonic stem cells (ESCs) [11], endothelial progenitor cells (EPCs) [12] and cardiac stem cells (CSCs) [13] (see Figure 1 for general properties of stem cells). Amongst the many choices available at present, MSCs have been the most favoured for the majority of published studies. MSCs are primarily isolated from the bone marrow, but they can also be derived from other tissue sources. Their popularity with researchers lies in a number of inherently advantageous properties, including easily identifiable cell surface markers, their adhesiveness to plastic facilitating culture *ex vivo* and their ability to differentiate into multiple cell lineages. Moreover, they can be sourced from an adult; hence, they are free from the ethical issues around research using ESCs [6]. More recently, pathfinder cells (PCs), a novel cell population named for their ability to navigate a path towards the site of damaged tissues *in vivo*, have been described [14-17].

Although there has been a great deal of discussion about how stem cells contribute to the regeneration of damaged tissues, much remains to be understood. One mechanism by which stem cells might repair damaged tissue is by means of differentiation of the stem cells into tissue-specific cell types [18,19]. Others have suggested that stem cells fuse with host cells in order to replace damaged tissue [20,21]. A more recent suggestion is that these cells work via paracrine signalling, instead of direct differentiation or fusion between cells. Paracrine signalling may act to stimulate damaged target cells to proliferate [22] or may induce other local cell types to differentiate [16]. The purpose of this review article is to provide an overview of the role that paracrine factors may play in various tissue damage models and how we can exploit this for the future treatment of disease.

### Paracrine action of stem cells in disease models

The idea that stem cells work via a paracrine action is now more widely accepted. Understanding paracrine signalling by cells involved in the repair of damaged tissue is important to the implementation of any future regenerative therapies. Paracrine signalling is defined as a form of communication between two different cells, where one cell releases chemical mediators to its immediate environment, which results in a change in the behaviour of a cell in its adjacent environment. There are numerous suggestions as to how the paracrine effects observed in many different model tissue repair systems are mediated. However, none has yet proven definitive.

The protective and regenerative effects of different stem cell therapies have been attributed to paracrine action in many cardiovascular studies. Coronary artery disease and heart failure are a leading cause of mortality and morbidity, particularly in the developed world. Consequently, many investigators have focused their efforts in this area of research. It has been proposed that paracrine effects contribute to the improvement of cardiac function following tissue insult and injury by modifying various factors such as inflammation, fibrosis, apoptosis, neovascularisation, contractility and cardiac repair [23].

A study that transplanted human cardiac progenitor cells (hCPCs) into a mouse model of myocardial infarction (MI), found that hCPCs isolated following transplantation using laser capture micro-dissection, had significantly increased expression of growth factors, such as vascular endothelial growth factor (VEGF), fibroblast growth factor 2 (FGF2) and connective tissue growth factor *in vivo*[24]. The transplanted hCPCs stained negatively for cardiac and endothelial differentiation markers, indicating that no differentiation of hCPCs had occurred [24]. Furthermore, MSCs and MSC-conditioned media enhanced cardiac excitation-contraction coupling [9]. The authors of this study suggested that this was solely mediated via a paracrine mechanism, attributed to a phosphoinositide-3-kinase (PI3K)/Akt induced change in calcium signalling and an endothelial nitric oxide synthase (eNOS) mediated change in sarco-endoplasmic reticulum calcium transport ATPase (SERCA) activity, and not by inter-communication between the MSCs and cardiomyocytes.

Additionally, one group [25] demonstrated that incubation of cardiomyocytes subjected to hypoxia and re-oxygenation with conditioned media from MSCs resulted in a cardio-protective effect. They suggested that this was due to a paracrine effect from the MSC-conditioned media, which protected the cells from damage by modulating the effect of a mitochondrial apoptotic pathway to reduce apoptosis via the inhibition of cytochrome C release from the mitochondria and by reducing activation of caspase-3 [25]. The protective effect of MSCs was also evident in a study using a rat model of pulmonary arterial hypertension (PAH). In this study [26], the authors demonstrated that sublingual vein injection of MSCs for two weeks resulted in improved lung and heart function following injury, improved pulmonary vascular remodelling, reduced inflammation and enhanced angiogenesis. These researchers suggested that these findings were possibly the result of a paracrine mechanism via secretion of anti-inflammatory mediators, as they observed reduced expression of inflammatory mediators, such as IL-1β, IL-6, TNF-α and matrix metallopeptidase-9 (MMP-9), but higher levels of VEGF, in their MSC-treated group compared to untreated groups [26].

Recently published work [27] has suggested a potential role for microRNAs in cardiac regeneration effects. These researchers found that adult rat cardiomyocytes with limited capacity for differentiation can be induced to enter the cell cycle and proliferate by the exogenous administration of microRNAs, hsa-mir-590 and hsa-miR-199a, in both *in vivo* and *in vitro* experimentation [27]. MicroRNAs are small RNAs of approximately 21 to 25 nucleotides that negatively regulate gene expression post-transcriptionally and which can affect the function of diverse biological processes [28]. MicroRNAs have been shown to be released by cells in small vesicles, such as microvesicles and exosomes, which will be discussed in more detail below.

The use of stem cells, particularly MSCs, in recovery from acute kidney injury (AKI) has also been extensively studied. Ischaemia/reperfusion (I/R) injury is known to cause delayed cellular regeneration and functional recovery following kidney transplantation [29]. It has been suggested that the reno-protective effect of administered MSCs in rats with I/R AKI was mediated primarily by the paracrine action of MSCs [30]. Significantly, these investigators found: (i) increased secretion of growth factors and upregulation of cytokines such as hepatocyte growth factor (HGF), VEGF, insulin-like growth factor-1 (IGF-1), IL-10, basic FGF, tumour growth factor alpha (TGF-α) and B-cell lymphoma-2 (Bcl-2), which are anti-inflammatory, anti-apoptotic and known to improve renal function; (ii) downregulation of pro-inflammatory mediators such as IL-1β, TNF-α, IFN-γ and inducible nitric oxide synthase (iNOS) and (iii) little to no intra-renal trans-differentiation events of administered MSCs [30]. Furthermore, they also demonstrated in a second study that MSC-conditioned media increased cell survival and the proliferation rate of endothelial cells *in vitro* and proposed that the vasculo-protective effect of MSCs was due to their ability to interact with endothelial cells by complex paracrine actions, which are able to protect and regenerate damaged vasculature in AKI significantly [7].

Wound healing is another area of research in which the possible effects of paracrine signalling by stem cells have been explored. Impairment of normal wound-healing processes often occurs in diabetic patients, leading to chronic wounds. These do not heal and can subsequently become gangrenous lesions or diabetic ulcers, often requiring the need for amputation. Thus, advancement of any regenerative therapy to repair such wounds and to promote fast healing would be beneficial. However, balance is needed between promoting fast wound healing and the formation of fibrous scar tissue, which could impair the function of the healed tissue or have a poor cosmetic appearance. One study [31] found that MSC-conditioned media accelerate wound-healing processes. Further investigations revealed that the medium contained high levels of growth factors and chemokines known to promote wound-healing. These included epidermal growth factor (EGF), keratinocyte growth factor (KGF), IGF-1, VEGF-α, erythropoietin (EPO), stromal cell-derived factor 1 (SDF-1), macrophage inflammatory protein (MIP)-1a and MIP-1b, suggesting that MSCs may work by a paracrine mechanism to accelerate wound healing [31]. MSCs have also been shown to release anti-fibrotic cytokines, which reduce the formation of scar tissue [32]. It has been suggested that foetal aorta-derived CD133^+^ progenitor cells (and a conditioned medium from their culture) also act similarly in a model of ischaemic diabetic-induced ulceration [33]. These researchers found that CD133^+^ cells accelerated wound closure and promoted angiogenesis via a paracrine effect through the release of cytokines, which affect the Wnt pathway, leading to stimulation of endothelial cell proliferation, migration and survival [33]. More recently, adipose-derived MSCs have also been used to demonstrate this effect in cutaneous wound healing [34].

The effect of paracrine secretion by stem cells has also been noted in experiments using MSCs to induce repair processes in spinal cord injury in rats [35]. Even after 21 days of observations, the MSCs had not directly become incorporated into the regenerated host tissue, though there was a significant improvement in functional recovery from as early as a week after MSC treatment, suggesting an MSC-mediated paracrine effect [35]. The use of MSCs in spinal cord injury has been extensively reviewed elsewhere by Wright *et al*. [36].

Recently, a study [16] showed regeneration of damaged adult pancreatic tissue and complete long-term functional recovery using a xeno-transplant model, whereby streptozotocin-induced diabetic mice were injected with rat or human PCs. PCs are a novel adult cell type, lacking the standard MSC markers CD105 and CD73 [15]. Crucially, the regenerated pancreatic tissue was not a result of the trans-differentiation of rat or human PCs into fully functional mouse pancreatic islets [16]. Instead, it was proposed that the recovery observed was more likely the result of a paracrine signal from the PCs [16]. Notably, this effect was not observed with the use of a PC-conditioned medium in this model, suggesting that it was not the result of cytokine or growth factor secretions, though this lack of efficacy might formally be due to a dose effect. PCs have subsequently been shown to repair renal damage resulting from ischaemic injury in a similar xeno-transplant model [17]. The low numbers of PCs or PC-derived cells found in the repaired kidney is again consistent with paracrine-mediated repair processes.

From the aforementioned experiments, it is evident that there is agreement amongst many researchers, tackling a diverse range of disease models, which clearly indicates the role that paracrine actions, as opposed to stem cell differentiation or cell fusion, have in repairing damaged tissues. Stem cells have been shown to target various local cells to exert their effects. For example, MSCs have been known to modulate the immune system by inducing immune cells, such as regulatory T cells, B-lymphocytes, natural killer (NK) cells and dendritic cells, and generating a regulatory phenotype of macrophages [37] while PCs affect pancreatic beta cells and are also immunomodulatory [16]. However, there still remains a need to further elucidate the precise mechanisms by which stem and progenitor cells initiate the repair of damaged tissues via paracrine actions. Aside from the observation that stem cells release a multitude of growth factors and cytokines to induce the reparative process, one exciting discovery is that these cells release microvesicles and exosomes, which may be a source of paracrine factors required for tissue repair and which may ultimately be used as therapeutic source material directly. Indeed, the recent discovery of a potential role for microRNAs, which occur in microvesicles and are one of the paracrine factors able to initiate repair processes in damaged cardiac tissue, adds another important dimension into the possibilities for therapeutic intervention [27].

### Microvesicles as mediators of paracrine effects

One of the most rapidly emerging ideas that explain paracrine mechanisms of tissue regeneration is the use of stem cell-derived micro-secretory vesicles, which act as mediators of tissue regeneration following injury or disease. This is an exciting area of research, as it opens up the potential to explore non-cell-based therapy in regenerative medicine.

Micro-secretory vesicles include microvesicles, which are one of a number of membranous vesicles derived from cells that were previously thought of as artifactual, resulting from cell preparatory methods or from cellular debris without any specific biological purpose. Recent evidence, however, has shown that microvesicles possess the ability to participate and influence numerous biological processes [38]. It is now generally accepted that microvesicles could aid in the transfer of genetic information between cells, as they contain proteins, messenger RNAs (mRNAs), DNAs and/or microRNAs. They also regulate the physiology and pathophysiology of cells and can be exploited for therapeutic and diagnostic purposes [38-41]. Thus, microvesicles could be a useful tool to treat solid organ damage as they may act as mediators to promote anti-inflammatory, pro-angiogenic, anti-apoptotic and differentiation or mitotic factors to activate the intrinsic repair and regeneration processes.

**Figure 2:**
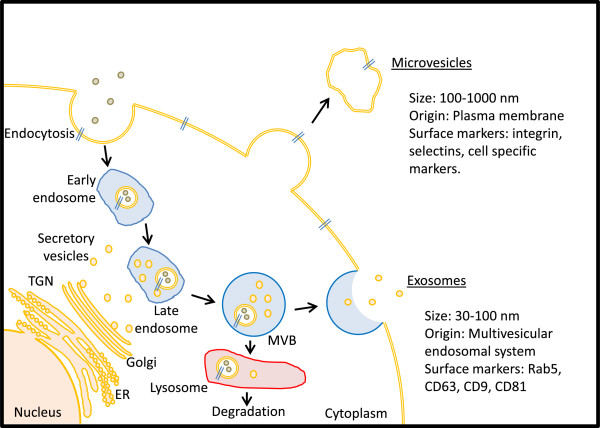
**Origin of microvesicles and exosomes from cells.** A microvesicle arises from budding of the plasma membrane. Microvesicles are more irregular in shape and size and can contain cytoplasmic materials. Microvesicles express surface markers such as integrin-β, CD40 and selectins such as plasma selectins and/or proteins from the cells they originate from. Exosomes originate from the endosomal trafficking system and, therefore, are more regular in shape and size. Exosomes are more easily identifiable via cell surface markers such as CD81, CD9 and CD63 and may contain materials such as mitochondrial DNAs, mRNAs and miRNAs. ER: endoplasmic reticulum; miRNA: microRNA; TGN: trans-Golgi network; MVB: multi-vesicular bodies.

There remains controversy surrounding the nomenclature for micro-secretory vesicles such as microvesicles and exosomes, based on their size and their isolation methodologies. Whilst agreement needs to be reached on a standard definition for these vesicles, it is widely understood and accepted that both of these types of vesicle are structurally and morphologically distinct (Figure 2). A microvesicle arises from budding of the plasma membrane of a cell. Microvesicles are generally more heterogeneous in terms of size, which can range from anywhere between 100 nm and 1,000 nm. Conversely, exosomes are derived from an endocytosis process within cells and are more homogeneous with respect to vesicle size, ranging from 30 nm to 100 nm [42,43]. It has been suggested that microvesicles will sediment at a lower centrifugation speed compared to exosomes, which sediment at 100,000* g*[43]. Since most studies tend to sediment membranes at 100,000* g*, any preparation will likely contain both microvesicles and exosomes, thus making it difficult to determine which of these contribute to the effects observed [43]. Furthermore, it should also be noted that most published research uses the term microvesicles to describe both of these types of micro-secretome. They are, however, distinct from apoptotic vesicles or bodies, which are shed from normal and diseased cells undergoing apoptosis [44].

Microvesicles derived from stem cells have already been shown to contribute to tissue and cellular regenerative processes. Microvesicles isolated from MSCs and EPCs have proliferative and anti-apoptotic effects on tubular epithelial and endothelial cells *in vitro*, in addition to being able to protect against AKI when delivered *in vivo* to a glycerol-induced severe combined immunodeficiency (SCID) mouse model [45] and to an I/R injury rat model [46,47]. This regenerative effect was also found to be specific for MSC- and EPC-derived microvesicles, as it was not observed with fibroblast-derived microvesicles. Moreover, the effect was abolished when the MSC- and EPC-derived microvesicles were treated with RNase prior to use, suggesting that a form of RNA is a key player in their reparative function [45,46].

This group of researchers also demonstrated the regenerative potential of microvesicles derived from human liver stem cells [48]. They showed the proliferative and anti-apoptotic properties of these human-derived microvesicles on human and rat hepatocytes *in vitro* and the regeneration and recovery of rat liver, when these microvesicles were administered into 70% hepatectomised rats [48]. They suggested that the microvesicles shuttle mRNAs involved in angiogenic pathways and microRNAs associated with cell proliferation, angiogenesis and inhibition of apoptosis, to the damage sites [45,46].

Additionally, recently published work [49] using microvesicles isolated from human umbilical cord MSCs, has demonstrated *in vitro* that microvesicles can be internalised by endothelial cells and promote the proliferation and angiogenesis of these cells in culture. Additionally, these researchers showed that this effect could be translated to *in vivo* experiments using a rat hind limb ischaemia model, whereby the exogenous microvesicles were found to be able to promote new blood vessel formation [49]. Further support for microvesicle/exosome-mediated paracrine repair processes comes from observations of the administration of MSC-derived exosomes to a murine model of hypoxic pulmonary hypertension (HPH). This resulted in suppression of the inflammatory processes, which are known to be a damaging factor in the development of HPH [50].

The combined results from all these data, although at a very early stage, have been encouraging in furthering our understanding of how stem cells, and in particular microvesicles, function in the body to initiate tissue repair. These observations have provided a tantalising insight into the possible use of microvesicles as a future cell-free therapy for tissue repair.

### Potential future treatment for regenerative medicine

Paracrine-mediated tissue repair might in future be exploited via genetic engineering of stem, progenitor or other cells to deliver various beneficial mediators to stimulate regenerative processes in damaged tissues. A number of researchers have looked at the potential of introducing beneficial factors by modifying stem cells to express these factors at the required level of efficacy. One such study [51] looked at using modified MSCs to express Wnt11; cardiomyocytes co-cultured with these MSCs had increased cell survival and reduced cell death following exposure to 40 hours of hypoxic conditions. In addition, MSC^Wnt11^ cell infusion *in vivo* improved cardiac function in rats following MI, as well as reducing apoptosis and fibrosis in the damaged hearts [51]. The authors suggested that this was because the release of Wnt11 caused regeneration of the damaged hearts, as Wnt signalling has previously been reported to promote cardiomyogenic repair [51]. A similar study employing MSCs engineered to overexpress Akt also successfully showed, *in vitro* and *in vivo*, the cytoprotective effects of these engineered cells on ischaemic hearts [52].

Perhaps one of the more exciting novel therapeutic approaches is the development of non-cell-based therapies for studying the effects that microvesicles derived from various types of cells have on tissue regeneration. Microvesicles as a source for drug therapy can be made naturally, from either modified or unmodified regenerative cells, as mentioned previously. This possibility was demonstrated recently in an *in vitro* study [53] where MSCs were modified to express cystinosin, the gene for which (CTNS) is mutated in a rare disorder called cystinosis, which causes a large accumulation of cysteine in cells and eventually results in cellular apoptosis. It was found that microvesicles isolated from CTNS-expressing MSCs also contained CTNS mRNA and when incubated with CTNS mutant fibroblasts ^(−/−)^, resulted in reduced cysteine accumulation in the cells [53]. Microvesicles may also be artificially manufactured to incorporate therapeutic entities for the treatment of a specific disease. This method may have certain advantages for the manufacturing processes and stringent quality control as well as because of the ability to design custom-made microvesicles that express proteins, mRNAs and/or microRNAs relevant to a particular disease or condition. Numerous ways in which microvesicles can be manipulated for use in regenerative medicine for organ and tissue regeneration and repair have been extensively reviewed by Ratajczak *et al*. [54].

## Conclusions

Although many details of cellular paracrine effects remain to be elucidated, it is clear that the potential for the discovery of novel therapies for regenerative medicine will rise exponentially in the not too distant future and may ultimately result in such therapies being transferred from the lab into the clinic. Indeed, this development is already becoming a reality as currently two groups of researchers have started phase 1 clinical trials using cardiosphere-derived cells [55] and cardiac stem cells [56] to improve patient outcomes following an episode of MI. The full results of the trials are very much anticipated by the rest of the science and medical communities.

## Abbreviations

AKI: Acute kidney injury; ASC: Adipose-derived stem cell; Bcl-2: B-cell lymphoma 2; CSC: Cardiac stem cell; EGF: Epidermal growth factor; eNOS: Endothelial nitric oxide synthase; EPC: Endothelial progenitor cell; EPO: Erythropoietin; ER: Endoplasmic reticulum; ESC: Embryonic stem cell; FGF2: Fibroblast growth factor 2; hCPC: Human cardiac progenitor cell; HGF: Hepatocyte growth factor; HPH: Hypoxic pulmonary hypertension; I/R: Ischaemia/reperfusion; IFN-γ: Interferon gamma; IGF-1: Insulin-like growth factor 1; IL: Interleukin; iNOS: Inducible nitric oxide synthase; KGF: Keratinocyte growth factor; MI: Myocardial infarction; MIP: Macrophage inflammatory protein; miRNA: MicroRNA; MMP-9: Matrix metalloproteinase-9; MSC: Mesenchymal stem cell; NK: Natural killer; PAH: Pulmonary arterial hypertension; PC: Pathfinder cell; PI3K: Phosphoinositide-3-kinase; SCID: Severe combined immunodeficiency; SDF-1: Stromal cell-derived factor 1; SERCA: Sarco-endoplasmic reticulum calcium transport ATPase; TGF-α: Tumour growth factor alpha; TGN: Trans-Golgi network; TNF-α: Tumour necrosis factor alpha; VEGF: Vascular endothelial growth factor.

## Competing interests

The authors declare that they have no competing interests.

## Authors’ contributions

DFA participated in the writing of the manuscript. PGS participated in its critical appraisal. Both authors read and approved the final manuscript.
